# Fear-focused Self-compassion Therapy for young breast cancer patients' fear of cancer recurrence: Study protocol of a randomized controlled trial

**DOI:** 10.3389/fpsyt.2022.941459

**Published:** 2022-09-23

**Authors:** Liuyu Wei, Xiaomin Yang, Shasha Sun, Yunlei Yu, Juan Xie, Jiang Zhao, Xiao Wang, Lei Zhu, Juntao Yao

**Affiliations:** ^1^School of Psychology, Shaanxi Normal University, Xi'an, China; ^2^Shaanxi Provincial Cancer Hospital, Xi'an, China

**Keywords:** fear of cancer recurrence, RCT, Fear-focused Self-compassion Therapy, young breast cancer patients, China

## Abstract

**Background:**

In China, there are a growing number of young women being diagnosed with breast cancer. Fear of Cancer Recurrence (FCR) has become one of the major psychological concerns reported by young breast cancer patients. Yet, there is a lack of psychological intervention tailored for reducing FCR for Chinese young breast cancer patients. In the current study, the Fear-focused Self-compassion Therapy is developed to help Chinese young breast cancer patients to manage FCR. The therapy was developed based on FCR development theories and self-compassion related therapy. The primary objective is to evaluate the short-term and long-term effectiveness of Fear-focused Self-compassion Therapy. The secondary objective is to examine the underlying mechanisms of therapy in reducing FCR in young breast cancer patients.

**Methods:**

The Fear-focused Self-compassion Therapy consists of 8-week face to face group sessions. This study will be a randomized controlled trial with 160 Chinese young female breast cancer patients with severe FCR. Participants will be randomized to the therapy group or a usual care control group (1:1). Measurements will be completed at baseline, immediately completing intervention, 3, 6, and 12 months later. Primary outcomes are FCR severity, and secondary outcomes include symptoms of depression and anxiety, satisfaction with therapy, and cost-effectiveness of the therapy.

**Discussion:**

If successful, this study will provide an effective psychological intervention to treat FCR for young breast cancer patients in China, and illuminate the underlying mechanisms of the Fear-focused Self-compassion Therapy in reducing FCR.

**Clinical trial registration:**

ClinicalTrials.gov: NCT04965428. Registered 23rd July 2021, https://clinicaltrials.gov/ct2/show/NCT04965428?cond=fear+of+cancer+recurrence&draw=2&rank=1

## Introduction

Globally, breast cancer has become one of the highly prevalent cancers in women (1). Similarly in China, the number of women being diagnosed with breast cancer is increasing, partly because of early screening and improved medical treatments (2). It is estimated that 0.37 million Chinese women are living with and beyond breast cancer in 2018 (2). About 20% of Chinese women with breast cancer are young people aged from 18 to 40 (3). Compared with older cancer patients, younger patients concern more about cancer recurrence or cancer progress (4–6). Especially for young breast cancer patients, they are more likely to have lymph node metastasis, an advanced cancer stage, a high histological grade, and a larger mass (7). Moreover, young breast cancer patients tend to choose breast conserving therapy for the consideration of future life (8). These can increase the risks of cancer recurrence and transference, which may cause clinically significant Fear of Cancer Recurrence (FCR). FCR has been found as one of the major psychological problems in young breast cancer patients (6, 9, 10). Up to 70% of young breast cancer patients suffer from clinically significant FCR (11). Therefore, it is of great clinical relevance to offer young breast cancer patients effective psychological interventions to address FCR.

FCR is defined as the fear and worry about cancer returning or cancer progress (12). FCR can be considered as a concern that may range on a continuum from mild and short-term normal fear to severe and long-term pathological fear (5). Mild FCR is adaptive, as it can prompt patients to be alert to their body symptoms (13), and increase patients' awareness of self-protection and adherence to treatment (14). Yet, severe pathological FCR may not only cause patients' overuse of primary care that increases unnecessary healthcare cost (15), but also lead to patients' intrusive thoughts, re-experienced trauma, hypervigilance, avoidance of cancer related memory, and difficulties in future planning (16, 17). This may negatively influence patients' quality of life, prognosis, and even survival (18).

Psychological interventions have been applied to reduce severe FCR in cancer patients (19–21). In particular, several RCTs have demonstrated the efficacy of psychological interventions for improving FCR, including mindfulness interventions (22–24), psychological education (25), cognitive behavioral therapy (26), and meta-cognitive therapy (27). Based upon previous empirical research, Tauber et al. conducted a meta-analysis and found that existing psychological interventions can improve FCR but to a small extent (Hedges's *g* = 0.28 – 0.33) (28). Moreover, only a few studies have tailored psychological interventions for young breast cancer patients to reduce FCR (21, 29). This calls for future studies to develop and test more effective interventions for addressing FCR (28).

To develop tailored psychological interventions to effectively address severe FCR, it is important to consider the development process of FCR into interventions. Up till now, previous research has developed several theoretical models explaining the development process of FCR including cognitive processing model (30), cognitive schema model of FCR (31), and cognitive-affective model (32). Although these models differed on components in the formulation of FCR, these models agree upon that cancer patients' cognitive evaluation about cancer recurrence plays a key role in the development of severe FCR (31). Specifically, when facing cancer-related stimulus in daily life (i.e., physical symptoms and anti-cancer advertisements), cancer patients are likely to have a dysfunctional cognitive evaluation, including catastrophizing of cancer recurrence, rumination of cancer-related traumatic memories and attentional bias to cancer related stimulus, which may cause FCR. Therefore, to help cancer patients to address severe FCR, it is needed to correct cancer patients' dysfunctional cognitive evaluation.

Self-compassion refers to treating oneself with kindness and warm when confronting with life stressors (33). Higher levels of self-compassion can help people relieve from pain and sufferings and cope with life traumas (e.g., cancer) with a tolerant, empathetic, and kind attitude (33). By treating oneself with kindness, people can adjust one's dysfunctional cognitive evaluations (e.g., rumination and catastrophizing) toward stressful events (34). There have been several empirical studies confirmed a link between high levels of self-compassion and low levels of rumination and catastrophizing (35, 36). Also, an experimental study has found that when inducing one's self-compassion with a writing task, people presented a greater ease in disengaging from negative stimuli, which clearly showed the impact of self-compassion on changing one's attentional process (37). Moreover, it is proposed that automatic nervous system (including the parasympathetic and the sympathetic nervous systems) plays an important role in self-compassion improving psychological wellbeing (38). Improved self-compassion has been found to relate to improved psychological symptoms through improved functioning of autonomic nervous system, with decreased salivary alpha amylase (i.e., indicator of sympathetic nervous system functioning) and increased heart rate variability (i.e., the index of parasympathetic nervous system functioning) (39, 40). Therefore, it can be hypothesized that improved self-compassion may lead to better psychological outcomes by adjusting one's attentional bias, dysfunctional cognitive evaluation, and the autonomic nervous system (i.e., the parasympathetic and the sympathetic nervous systems).

More recently, self-compassion related therapy (such as compassion-focused therapy and mindful self-compassion therapy), aiming to enhance self-compassion and help individuals better cope with stressful life events (38, 41), has been shown to increase the levels of self-compassion (Hedges's *g* = 0.75), and reduce symptoms of depression (Hedges's *g* = 0.66), anxiety (Hedges's *g* = 0.57), and stress (Hedges's *g* = 0.67) (42). With unique Eastern Buddhist characteristics, self-compassion related therapy involves a part of mindful meditation and aims to encourage individuals to keep self-kind and self-understanding to relieve stress when facing stressful events.

In the context of cancer, the beneficial role of self-compassion on psychological outcomes (including symptoms of depression, anxiety, and fatigue) has been confirmed in Chinese cancer patients (43, 44). As for FCR, intervention studies have started to examine the feasibility of self-compassion therapy in the application of coping with FCR (45, 46). Using randomized controlled trails, two studies focused on middle aged and elderly breast cancer patients, and confirmed that an 8-week self-compassion intervention was feasible and acceptable for these patients to decrease levels of FCR by enhancing one's self-compassion (45, 46). Yet, given the inclusion of middle aged and elderly breast cancer patients from the Western countries and the use of small sample size, it remains unclear to what extent their conclusions can be generalized into young breast cancer patients in China. Moreover, the two previous studies only examined the short-term (i.e., no longer than 6 months) efficacy of self-compassion related therapy on FCR, and it remains unknown about the long-term (i.e., longer than 6 months, such as 12 months after intervention) effectiveness and why self-compassion related therapy would be effective in reducing severe FCR.

### Aims of this study

In the current study, the Fear-focused Self-compassion Therapy, aiming to help Chinese young breast cancer patients to address FCR, has been developed. The primary aim of the current randomized controlled trial is to evaluate the effectiveness (i.e., both short-term and long-term effectiveness) and cost-effectiveness of Fear-focused Self-compassion Therapy in reducing severe FCR in young women with breast cancer, compared to usual care. Due to a lack of research comparing the cost-effectiveness between compassion-based therapy and usual care, it is hard to hypothesize which condition would be more cost-effective. This study expects that Fear-focused Self-compassion Therapy would be more cost-effective than usual care by reducing patients' FCR more effectively. Several specific hypotheses were outlined:

1. FCR severity would reduce to a larger extent in the Fear-focused Self-compassion Therapy condition (intervention group) compared to the treatment as usual condition (control group) from baseline and follow-up (3 months after treatment). The improvements in FCR can maintain at least 6 months after treatment.
2. Symptoms of depression and anxiety would reduce to a larger extent in the Fear-focused Self-compassion Therapy condition compared to the treatment as usual condition from baseline and follow-up (3 months after treatment). These effects can maintain at least 6 months after treatment.
3. Fear-focused Self-compassion Therapy would be cost-effective compared to treatment as usual.

The secondary aim of this study is to examine the underlying mechanisms of Fear-focused Self-compassion Therapy in reducing severe FCR. Specifically, the current research assumed that patients' improved dysfunctional cognitive evaluations and improved functioning of autonomic nervous system would play mediating roles between Fear-focused Self-compassion Therapy and improved FCR. Specific hypotheses are:

1. Fear-focused Self-compassion Therapy can increase cancer patients' levels of self-compassion, then improve cognitive evaluation processes (i.e., decreased catastrophic expectation to cancer recurrence, less attentional bias toward cancer-related stimulus, and less rumination), and reduce levels of FCR.
2. By improving levels of self-compassion, Fear-focused Self-compassion Therapy could improve the functioning of sympathetic nervous system (with decreased salivary alpha amylase as physiological index) and parasympathetic nervous system (with increased heart rate variability as physiological index), and decrease levels of FCR.

## Methods

This study will be a randomized controlled trial, in accordance with the CONSORT statement for psychological interventions and the SPIRIT 2013 statement (47, 48). This study was funded by National Natural Science Foundation of China and Project of Humanities and Social Sciences, and registered in the clinicaltrial.gov (Clinical Trial No. NCT04965428, date assigned 23/07/2021). Ethics approval has been obtained from Ethics Committee of Shaanxi Provincial Cancer Hospital.

### Study design

The current study will compare Fear-focused Self-compassion Therapy with usual care in young women with breast cancer. A sample of 160 young female breast cancer patients with clinically significant FCR will be recruited from Shaanxi Provincial Cancer Hospital in Xi'an, China. After checking the eligibility of participants and completed the baseline measurement (T0), they will be randomized to either the Fear-focused Self-compassion Therapy or usual care control group. Four follow-up measurements will take place for both groups: immediately completing intervention (T1), after 3 months (T2), after 6 months (T3), and after 12 months (T4).

### Participant eligibility

Women are eligible to participate if they: (1) are diagnosed with breast cancer (including various cancer stages and subtypes); (2) are aged 18–45 years; (3) can sign written informed consent; (4) can read and write Chinese; and (5) have clinically significant FCR, with scores ≥13 on the FCRI severity subscale (49).

If participants have a cancer recurrence during the study, they would remain in the therapy group, but their data would not be included in the final data analysis. Women are ineligible to participate if they: (1) currently have psychiatric and psychological illness; (2) have other types of tumor; (3) had a previous cancer recurrence; and (4) are participating another psychological intervention at the start of the study or during their 8-week therapy.

### Recruitment settings and procedure

Participants of the current study will be recruited at Shaanxi Provincial Cancer Hospital in Xi'an, China. We will place recruitment posters in noticeable places (e.g., waiting areas and elevators for patients) in hospital. Oncology nurses will also be involved to inform eligible patients about this study during their check-up at the outpatient clinic, and ask patients if they would be interested to participate in this study. Research assistants will contact patients whom are interested in the study to explain more about the study, and screen patients for eligibility. After confirmation of eligibility, patients who agree to participate in the study will receive an informed consent.

### Randomization

A table of randomized number will be generated by using the SAS. According to the table of randomized number, participants will be randomized to the intervention or control group with an allocation ratio of 1:1, after stratified by age, cancer stage, and breast cancer subtype. Participants will not know which group they will be allocated in.

### Intervention

The theoretical frame of the Fear-focused Self-compassion Therapy is based on the theories of FCR development as well as the theories of self-compassion related therapy such as compassion-focused therapy and mindful self-compassion therapy (31, 33, 38, 50, 51). The Fear-focused Self-compassion Therapy is an 8-week compassionate mind training intervention, and will be delivered as a group format. It is developed and tailored for Chinese young breast cancer patients by the authors including clinical psychologists, cancer nurses, and oncologists, adapted to be appropriate for Chinese young breast cancer patients and tailored to FCR. The therapy consists of eight group face-to-face sessions (60–90 min for each session) and will be delivered by three trained therapists for 8 weeks (sessions are weekly). The therapy will take place in Shaanxi Provincial Cancer Hospital, and each intervention group involves 10–15 patients. The content outline of intervention is shown in Table 1.

**Table 1:** Content of fear-focused self-compassion therapy sessions.

**Session**	**Theme**	**Aims**	**Intervention content**
1.	The stability of attention	- Learn how to control the attention; - Recognize cancer-related thinking and worry; - Decrease over-identification.	- Introduce the plan, structure, confidentiality, and safety of intervention; - Introduce Fear-focused Self-compassion Therapy's theory; - Set the goals of intervention; - Introduce the attention; - Recall the experience of thinking in “automatic drive”; - Mindfulness meditation; - Discuss and set the aims, plans, and problems that may occur in home practice.
2.	Insight	- Observe your cancer-related thoughts and emotions; - Recognize your emotions and behaviors reacting to worry about cancer recurrence; - Experience the peace derived from the observation and non-involvement to the thoughts of cancer recurrence.	- Share your home practice and problems; - Deal with the problems left from last week; - Positive and negative thoughts, sensory training; - Stress response training; - Mind rest training; - Mindfulness meditation; - Image breath practice; - Discussion; - Renew the goals of home practice.
3.	The heart of self-compassion	- Understand FCR is not the direct effect of external condition, but the result of dysfunctional cognition (i.e., catastrophizing thinking, rumination, and attentional bias); - Recognize that FCR can be transformed; - Realize that you have the ability to transform and release from the fear.	- Discuss the home practice; - Introduce “Self-Compassion” and understand its components (recognize the cause of worry about cancer recurrence; realize that you have the ability to make a change; make up your mind to change); - Analyze the difference between self-compassion and self-reception; - Mindfulness meditation; - Discussion.
4.	Impartial attitude	- Decrease the absolute classification for friends, enemies, and strangers; - Understand that classification of people with their utility will hinder them from building up relationship; - Realize that everyone pursues happiness and avoids pain, and develop an impartial perspective; - Learn not to pay too much attention to cancer-related stimuli in your life.	- Discuss the home practice; - “I care/I do not care” training; - Mindfulness meditation; - Image breath practice; - Discussion.
5.	Learn appreciation and gratitude	- Recognize that your “independent” life comes from others' support; - Develop the appreciation and gratitude toward others; - Further enhance the connection with others.	- Discuss the home practice; - A network of interdependence; - Discuss “gratefulness”; - Recall the kindness from others; - Image breath practice; - Discussion.
6.	Develop empathic	- Express stronger sensibility and sympathetic for others' misfortune and happiness; - Decrease the egocentricity.	- Discuss the home practice; - Share the research of empathic; - Discuss the psychological and physiological problems about loneliness, and the desire for connection; - Discuss the empathic and accidie; - Mindfulness meditation; - Discussion.
7.	Be hopeful, love, and compassion to life	- Understand that everyone pursues happiness and avoids pain, on the basic of love and empathic; - Recognize your consistence with others; - Enhance the connection with others.	- Discuss the home practice; - Reflect on others, specifically to difficult individuals, think about how to connect with their sufferings; - Mindfulness meditation; - Image breath practice; - Discussion.
8.	Be love and compassion with commitment	- Make a commitment to future life.	- Discuss the home practice; - Review the theory of self-compassion practice; - A summary of previous practice; - Set goals for home practice at the end of the intervention.

At the first session of the therapy, participants will obtain a pamphlet about the framework and goals of Fear-focused Self-compassion Therapy, as well as activities of each session. At the end of each session of the therapy, relevant home practice will be given to participants, and at the beginning of next session, these home practice will be reviewed to discuss participants' experience during home practice. When completing the therapy, participants will receive a handbook including the content of Fear-focused Self-compassion Therapy.

To make sure that all participants receive the same treatment, new participants should not be involved in the group if the intervention starts. Also, all participants are told that if they are absent for more than one session, they have to drop out this group and can choose other groups that has not started.

### Usual care

Participants in the control group will receive usual care supported by the hospital or coming from elsewhere, and will not be offered psychological intervention for addressing FCR. Care as usual will be accessible for patients in both intervention group and the control group. Their use of usual care will be measured by Medical Consumption Questionnaire.

### Outcomes

There are five measurement time-points for all participants in the intervention group and usual care control group as above mentioned. Patients' sociodemographic and clinical characteristics (i.e., breast cancer stage, education, income, socioeconomic status, and marriage) will be assessed at baseline (T0) only. Measurement time-points of all outcomes are shown in Table 2.

**Table 2:** Measurement time point.

**Time point**	**Instrument**	**Baseline (T0)**	**Immediately completing intervention (T1)**	**After 3 months (T2)**	**After 6 months (T3)**	**After 12 months (T4)**
Socioeconomic outcomes		√				
**Primary outcomes:**
Fear of cancer recurrence	FCRI	√	√	√	√	√
**Secondary outcomes:**
Salivary alpha amylase	Nipro salivary alpha amylase analyzer	√	√			
HRV	MP150-Biopac data acquisition system	√	√			
Attentional bias for FCR	The dot-probe task	√	√			
Rumination	CERQ-RS	√	√			
Catastrophizing	CERQ-CS	√	√			
Psychological symptoms	HADS	√	√	√	√	√
Self-compassion	SCS	√	√	√	√	√
Quality-adjusted life year	EQ-5D	√	√	√	√	√
Healthcare consumption	MCQ	√	√	√	√	√
Process outcomes			√			

#### Primary outcomes

Fear of cancer recurrence will be assessed using 42-item Fear of Cancer Recurrence Inventory (FCRI). This questionnaire consists of seven subscales, with a total score representing one's levels of FCR. A score of 13 or higher on 9-item severe subscale (ranges from 0 to 36) indicates a clinical FCR. The FCRI has been shown to have a good validity (convergent validity *r* = 0.68–0.77) and reliability (Cronbach's *alpha* = 0.95) (49).

#### Secondary outcomes

Neurophysiological data include *salivary alpha amylase* and *heart rate variability (HRV)*, and will take about 10 min to measure. The salivary alpha amylase activity will be measured by using a handheld salivary alpha amylase monitor manufactured by Nipro (Osaka, Japan). This analyzer can automatically measure the salivary alpha amylase activity within 1 min (about 30 s for saliva collection and 30 s for analyzing). Participants will be asked to brush teeth before measurement and not allowed to intake any food, beverage, tobacco or liquor within an hour. The HRV will be measured by using signal detection and amplification system (Biopac ECG100C) of MP150-Biopac data acquisition system. The Electrocardiogram (ECG) will be assessed with Lead-II configuration to measure participants' heart rate. The BiopacRSP100C will be used to collect respiratory rate, with filter rate ranges from 35 to 0.5 Hz. The CardioBatch will be used to analyze the ECG and respiratory rate, and compute the HRV. Participants will be asked not to intake any stimulant (e.g., caffeine and alcohol), or cannot do any strenuous activity within 4 h before measurement.

*Attentional bias for FCR* will be assessed by conducting the dot-probe task, including 200 trials with the stimuli of cancer-related words and utilized in previous study (52). The dot-probe task will be presented on a laptop computer and run by using Matlab. At the beginning of each trial, a “+” will present for 500 ms in the center of the screen. After that, a pair of word stimuli with a neutral word (e.g., apple) and a cancer-related word (e.g., chemotherapy) will be presented for 500 ms on the left and right sides of the screen. Then, the probe stimuli (i.e., “^*^” and “^**^”, representing the left and right button of the keyboard, respectively) will be presented in the position where the word stimuli just appear. Once the probe stimuli appear, participants should press button of the keyboard as soon as possible. After that, the screen will present 500 ms of blanking, and the next trial will start. Participants' reaction time to probe stimuli will be used to assess their attentional bias to cancer-related words. Cancer-related words are derived from a previous study examining attentional bias (52).

*Rumination* will be measured by using the 4-item Cognitive Emotion Regulation Questionnaire Rumination Subscale (CERQ-RS). Each item of CERQ-RS ranges from 1 to 5, and this subscale has been shown to have a good reliability with Cronbach's *alpha* of 0.83 (53).

*Catastrophizing thinking* will be measured by using the 4-item Cognitive Emotion Regulation Questionnaire Catastrophizing Subscale (CERQ-CS). Each item of CERQ-CS ranges from 1 to 5, and this subscale has been shown to have a good reliability with Cronbach's *alpha* of 0.79 (53).

*Psychological symptoms* will be measured by using Hospital Anxiety and Depression Scale (HADS). This 14-item questionnaire consisting of both anxiety subscale and depression subscale, rates on four-point (1–4) scales, and has been shown to have a good validity and reliability (54).

*Self-compassion* will be measured by using the 26-item Self-compassion Scale (SCS) consisting of six facets: self-kindness (5 items), self-judgment (5 items), common humanity (4 items), isolation (4 items), mindfulness (4 items), and over-identification (4 items) (55). Responses were rated on a five-point scale from 1 (*almost never*) to 5 (*almost always*). There is adequate evidence that SCS is valid and reliable (55).

The economic evaluation will be assessed with EuroQol-5D (EQ-5D). The EQ-5D includes five dimensions: mobility, self-care, usual activity, pain/discomfort, and anxiety/depression. Each dimension is assessed by one question with three levels of answers: no problem, some/moderate problems, and extreme problems. A health state can be obtained from a 5-digit number by combining the scores of each dimension. For instance, state 11,233 would indicate no problem with mobility or self-care, some problems with usual activities, and extreme problems with pain/discomfort, and anxiety/depression. The EQ-5D health states can be converted into an EQ-5D index to evaluate Quality-Adjusted Life Year (QALY). The EQ-5D has been widely used in cost-effectiveness analyses in various of clinical areas (56).

The *healthcare costs* of cancer patients in this study will be assessed with Medical Consumption Questionnaire (MCQ). It has been shown as a feasible and reliable instrument to measure medical consumption in patients with mental health problems (57).

#### Process outcomes

The process outcomes include participants' intervention adherence, satisfaction and acceptability. These outcomes will be assessed through self-report questionnaires completed by participants, and checklists and notes completed by researchers and therapists during the intervention.

### Cost-effectiveness analyses

The economic evaluation includes a cost-effectiveness analysis and a cost-utility analysis, both done from societal perspective. The cost-effectiveness analyses will calculate any incremental cost of each participant obtaining a significant decrease on FCRI Severity score (0.5 of the SD in FCRI Severity subscale is identified as a meaningful change) in both intervention and control groups (58). A lower incremental cost of each participant gaining a significant decrease on FCRI Severity score means higher cost-effectiveness of the group than another group. The cost-utility analysis will evaluate any incremental cost of each participant gaining a Quality-Adjusted Life Year (QALY) in therapy group. QALY will be calculated by using EQ-5D to measure Health-Related Quality of Life (HRQoL) weights (56).

The costs will be estimated from a societal perspective, and includes the costs related to therapy (excluding development or research costs), all healthcare costs and non-healthcare costs during the therapy. The therapy costs will be estimated based upon research team and hospital records (screening costs, delivery costs, and material costs of intervention). Other costs (i.e., healthcare costs and non-healthcare costs) will be estimated according to a resource use diary. The diary can be used to record patients' health service use (e.g., health consultations, hospitalization, emergency room visits, medication use, other psychological therapies use, and self-monitoring behaviors). The healthcare costs will be measured by Medical Consumption Questionnaire (MCQ) (57). Uncertainty in the cost and outcome will be estimated by non-parametric bootstrap analysis. The sensitivity analysis will be used to test the robustness of the economic evaluation results.

### Sample size calculation

The sample size calculation is based on a previous psychological intervention research aimed at reducing breast cancer patients' FCR, with an effect size of *d* = 0.49 (46). The power analysis program G^*^Power was used to calculate the sample size. At least total 134 participants (67 participants in each group) need to be included to detect an effect of *d* = 0.49 with a power of 0.8 and two-side *alpha* level of 0.05. Considering the dropout of participants, we added 20% of the sample size. Therefore, this study needs a total of 160 participants, with 80 participants in each group.

### Statistical analyses

The aim of the primary analysis is comparing the Fear-focused Self-compassion Therapy group with usual care control group on the score of FCRI severity subscale. In the primary analysis, post-treatment scores will be compared between two groups at four measurement time points (T1, T2, T3, and T4). Analysis will be performed by using linear mixed models, including baseline scores, sociodemographic and medical variables as covariates.

The aim of secondary analysis is to test the mediating role of improved dysfunctional cognitive evaluation and improved functioning of autonomic nervous system between Fear-focused Self-compassion Therapy and improved FCR. Analysis will be performed by using multilevel structural equation model, including baseline scores, sociodemographic and medical variables as potential covariates.

## Discussion

This RCT will be conducted with two aims. The primary aim is to evaluate the short-term and long-term effectiveness and cost-effectiveness of Fear-focused Self-compassion Therapy for reducing severe FCR in young women with breast cancer. The secondary aim is to examine whether improved dysfunctional cognitive evaluation and improved functioning of the autonomic nervous system would play a mediating role between Fear-focused Self-compassion Therapy and improved FCR.

### Strengths and weaknesses

This RCT of Fear-focused Self-compassion Therapy has several strengths. First, given that most of previous research focused on middle aged and elderly cancer patients, this study could fill the knowledge of the application of Fear-focused Self-compassion Therapy in young breast cancer patient population. Second, this study examines the long-term effectiveness of Fear-focused Self-compassion Therapy for patients' FCR, to verify whether this intervention is valid for controlling FCR in a long period of time. Third, by verifying the mediating role of dysfunctional cognitive evaluation and autonomic nervous system, this study will find the underlying mechanisms of Fear-focused Self-compassion Therapy for FCR, and provide implication for future research to develop more targeted intervention. Finally, this study will promote the implementation of Fear-focused Self-compassion Therapy for patients' FCR in Chinese background and investigate whether Chinese cancer patients could benefit from this therapy. However, in the present study without the third group with other intervention, it is not allowed to compare the effectiveness of Fear-focused Self-compassion Therapy with other intervention (i.e., mindfulness-focused therapy and cognitive behavior therapy).

Considering the lack of intervention research for young breast cancer patients, this study will contribute to the knowledge about how to decrease FCR in this population. Research into long-term effective psychological treatment for this population will be of great clinical value.

## Ethics statement

The studies involving human participants were reviewed and approved by Shaanxi Provincial Cancer Hospital Ethics Committee. The patients/participants provided their written informed consent to participate in this study.

## Author contributions

LW, SS, and YY drafted the manuscript. XY, JX, JZ, and XW will be involved in recruiting participant. LZ and JY initiated and supervised the project. All authors contributed to the study design, protocol development, and approved the final version of this manuscript.

## Funding

This study was funded by the National Natural Science Foundation of China (Award number: 32000773) and Project of Humanities and Social Sciences (Award number: 20YJA190013), with no influence on design, collection, analysis or interpretation of data, or writing the manuscript.

## Conflict of interest

The authors declare that the research was conducted in the absence of any commercial or financial relationships that could be construed as a potential conflict of interest.

## Publisher's note

All claims expressed in this article are solely those of the authors and do not necessarily represent those of their affiliated organizations, or those of the publisher, the editors and the reviewers. Any product that may be evaluated in this article, or claim that may be made by its manufacturer, is not guaranteed or endorsed by the publisher.

## Abbreviations

FCR: Fear of cancer recurrence
RCT: Randomized controlled trial
FCRI: Fear of cancer recurrence inventory
SAS: Statistical analysis system
SCS: Self-compassion Scale
SCS: Self-compassion Scale
HRV: Heart rate variability
ECG: Electrocardiogram
CERQ-RS: Cognitive Emotion Regulation Questionnaire Rumination Subscale
CERQ-CS: Cognitive Emotion Regulation Questionnaire Catastrophizing Subscale
HADS: Hospital Anxiety and Depression Scale
QALY: Quality-adjusted life year
EQ-5D: EuroQol-5D
HRQoL: health-related quality of life
MCQ: Medical Consumption Questionnaire.
